# Open label placebo to treat fatigue in people with multiple sclerosis: feasibility and preliminary effects

**DOI:** 10.1186/s40814-025-01674-w

**Published:** 2025-07-03

**Authors:** Navneet Kaur Baidwan, Tracy Tracy, Chia-Ying Chiu, Tanjila Nawshin, Teri Hoenemeyer, Emily Riser, Robert Motl, Kevin Fontaine, Tapan Mehta

**Affiliations:** 1 Department of Family and Community Medicine, Heersink School of Medicine, University of Alabama at Birmingham, Birmingham, USA; 2 Tanner Center for Multiple Sclerosis, Birmingham, AL USA; 3 Comprehensive Cancer Center, University of Alabama at Birmingham, Birmingham, USA; 4 Department of Neurology, Heersink School of Medicine, University of Alabama at Birmingham, Birmingham, USA; 5 Department of Kinesiology and Nutrition, College of Applied Health Sciences, University of Illinois Chicago, Chicago, USA; 6 Department of Health Behavior, School of Public Health, University of Alabama at Birmingham, Birmingham, USA

**Keywords:** Multiple sclerosis, Fatigue, Open-label placebo

## Abstract

**Background:**

Fatigue is highly prevalent in adults with multiple sclerosis (MS) and current treatments offer limited benefit. It has been speculated that placebos only have an effect when they are administered with deception and concealment, which is unethical in clinical practice. Recent studies suggest that ethically informed, open-label placebos (OLP) can produce symptomatic benefits. As such, we primarily sought to investigate the feasibility and secondarily assess the preliminary effects of OLP to treat MS fatigue.

**Methods:**

Feasibility outcomes including accrual, retention, and OLP adherence estimates were assessed in this 21-day assessor blinded, RCT. We compared results of assignment to three conditions: (1) OLP with a positive expectancy for beneficial effects along with the prescription to take 2 placebo pills twice a day (OLP), (2) positive expectancy (EXP) for beneficial placebo effects, or (3) a usual care only (UCO). We considered the study to be feasible if progression criteria, including the enrollment target of 48 participants, retention target of > 80% participants, and OLP adherence target of > 90%, were met. As secondary analyses, we provide descriptive statistics and crude linear mixed models (LMM) based estimates to assess the change in fatigue (assessed via Fatigue Severity Scale (FSS) and Modified Fatigue Impact Scale (MFIS)) at days 21, 28, and 35 versus baseline with corresponding 95%, 85%, and 75% confidence intervals.

**Results:**

One-hundred and eight adults with MS were screened of which 48 were randomized (16 per group). Retention rate was 98% with one participant being lost to follow-up. Placebo adherence was over 90%. At day 21, 7 of 9 (78%) randomized to OLP considered prescribing placebos to treat fatigue as “moderately-to-completely” acceptable. Next, the LMM based change in FSS mean score at day 21 with respect to baseline in the OLP and EXP group versus UCO group was about 0.6 units lower (95% CI: − 1.206, − 0.003; − 1.301, − 0.065, respectively).

**Conclusions:**

OLP was deemed feasible and acceptable by most participants and there was mild evidence that, compared to UCO, it may reduce fatigue severity in adults with MS. Larger trials of OLP are required to determine whether OLP might be a viable treatment for MS fatigue.

**Trial registration number:**

NCT04002102 (https://clinicaltrials.gov/show/NCT04002102, 2019); registered 30 September 2019.

**Supplementary Information:**

The online version contains supplementary material available at 10.1186/s40814-025-01674-w.

## Key messages regarding feasibility

What are the key feasibility findings?

- What uncertainties existed regarding the feasibility?
  Feasibility of OLP (open-label placebo) in MS-related fatigue had not been studied before. It was unclear whether conducting the protocol would be feasible and the intervention considered to be acceptable.
- What are the key feasibility findings?
  OLP (OLP with a positive expectancy for beneficial effects along with the prescription to take 2 placebo pills twice a day) was deemed feasible and acceptable by most participants and preliminary evidence suggests that, compared to UCO (usual care only), it may reduce fatigue severity in adults with MS.
- What are the implications of the feasibility findings for the design of the main study?
  Effect estimates obtained from this study can be used to design a future confirmatory trial which can manipulate expectancies (e.g., neutral vs. positive) and practitioner-patient relationship factors (e.g., empathy, active listening) to test the efficacy of OLP in treating MS-related fatigue.

## Background

Multiple sclerosis (MS) is a chronic, neuro-inflammatory disease of the central nervous system [1, 2].

Vast majority (75–95%) of adults with MS report having fatigue and identify it as a burdensome symptom disrupting daily life, personal relationships, and quality of life [2–4]. Fatigue in MS has been linked to longer durations to disease, increased disability, and progressive subtypes of MS [5]. Fatigue is a self-reported, subjective symptom without a unified definition without a gold standard to measure it. It may include exhaustion, lack of energy, or tiredness, and “a subjective lack of physical and/or mental energy that is perceived by the individual or caregiver to interfere with usual or desired activity” [6]. Current treatments, including pharmacological, sleep regulation, and psychological/behavioral interventions, have been of limited benefit, with there being no FDA-approved medication for fatigue in MS [2, 7].

Our central premise is that placebo pills may be beneficial for managing fatigue in MS. Placebo pills match active pharmaceuticals in appearance, but have no apparent physiological effects on symptoms, and yet produce benefits beyond those which could be explained by spontaneous improvement, regression to the mean, or the normal waning of symptoms [8]. Placebo effects are believed to operate via aspects of the therapeutic encounter (e.g., attention, warmth, empathy from provider), accompanied with symbols (e.g., white coats), rituals (e.g., taking pills), expectancies (e.g., “medication can make me feel better”), and hope (e.g., “there are still possibilities”) [8].

The possibility of producing a therapeutic effect using placebos without concealment and deception was first demonstrated in 1965 by Park and Covi [9], yet the gold standard for pharmaceutical RCTs remains the deceptive use of placebos as a comparator to an active compound. In such trials, patients in double-blind placebo conditions often experience significant improvement in outcomes. Although placebos were long assumed to confer benefits only when given with concealment or deception, a groundbreaking 2010 study [10] and over a dozen subsequent RCTs show that non-deceptive, open-label placebos (OLP) significantly relieved symptoms in several conditions such as irritable bowel syndrome [10], migraines [11], chronic back pain [12, 13], allergic rhinitis [14], and menstrual hot flashes [15], even when prescribed with honesty and transparency. Existing empirical findings [16] also support the claim that patients consider open placebos to be ethical and appear satisfied that open placebos are acceptable. It has been further concluded that open placebos fulfill current American Medical Association guidelines for placebo use and harnessing the placebo effect without placebos may provide an ethical, non-deceptive means of treating patients effectively [16].

We have previously conducted a 21-day assessor blinded RCT comparing OLP with treatment as usual for cancer-related fatigue [17]. To the best of our knowledge, no previous effort has assessed the potential effect of OLP on MS-related fatigue. However, we have examined OLP for cancer-related fatigue (CRF) where we conducted a 21-day, single site, two-parallel arm RCT to compare the effects of OLP to treatment as usual among cancer survivors reporting at least moderate CRF. The OLP treatment produced a 29% reduction in fatigue severity (*p* = 0.008, *d* = 0.63) and a 39% reduction in the extent to which fatigue disrupted quality of life (*p* = 0.002, *d* = 0.76) [17]. Similar findings on OLP’s effects on CRF were obtained in two independent replication RCTs [18, 19] with effect sizes on fatigue ratings of 0.57 and 0.70, respectively. These results provide evidence for the potential value of using OLP to also treat MS fatigue.

Therefore, we conducted a pilot study to primarily investigate the feasibility and secondarily assess preliminary effects of OLP to treat MS fatigue. Note that, the goal of feasibility studies is not to test hypotheses but to assess if refinements to the intervention would be needed for a full scale RCT, and to assess and address potential uncertainties pertaining the feasibility of intervention trial methods, and/or test preliminary effects of the intervention [20]. Pilot studies are not powered to assess effect [21] and the results for the secondary outcomes should be interpreted with caution.

## Methods

### Design and participants

Between 8/28/2020 and 12/22/2021, we conducted a 21-day, single site, three-parallel arm RCT among adults with MS reporting at least moderate fatigue to compare the effects of [1] OLP (i.e., prescription to take 2 placebo pills, twice a day) along with conveying positive expectations for effectiveness, [2] positive expectancy for placebo effects alone (EXP) with a breathing log and instructions to breathe for 1 min in the morning and evening, with the ritual mimicking that of OLP group, and [3] UCO only (i.e., standard care and educational materials). The intent here was to compare OLP that comprises setting expectations with the ritual of taking a pill versus only EXP where we separated the effect of the pill ritual, along with comparing both OLP and EXP with UCO. Block randomization with block sizes of 3 and 6 were used to randomize participants to the three arms. The choice of 21 days is based on prior OLP studies [10, 17, 22], as well as being within the range of the intervention period used in MS fatigue pharmacological trials. Adults with MS were recruited from Tanner Foundation, a regional clinic treating complex neurological diseases located in Birmingham, AL, USA. The Institutional Review Board (IRB) of the University of Alabama at Birmingham (UAB) approved this study, and written informed consent was obtained from each participant.

Participants were included if they were older than 19 years, had a confirmed diagnosis of MS, and reported at least moderate (≥ 4 on the 0–7 scoring of the Fatigue Severity Scale and a score of ≤ 7 on the Patient Determined Disease Scale) [23]. Patients on disease modifying therapies (DMTs) such as injectables or oral medications were enrolled if these DMTs were administered at a stable dosage for at least 90 days. We excluded participants with major comorbid conditions that might influence fatigue such as lupus and chronic fatigue syndrome. We also excluded those being treated with off-label medications or those who had participated in an exercise program in the past 30 days.

Patients referred by a neurologist were screened by the clinical coordinator, and if the individual reported moderate-to-severe fatigue, they were told they were eligible to participate in “a new mind–body study to treat fatigue in adults with multiple sclerosis.” If interested, a virtual clinic visit (via the encrypted version of Zoom) was scheduled for the individual. After completing the informed consent and baseline assessments, all participants were randomized into one of the three arms of the study with all assessments being done virtually.

Sixteen participants each were then randomized into one of the 3 arms (OLP, EXP, or UCO), using block randomization. All participants randomized to the OLP and EXP arms met for 15 min with the care provider who followed a scripted orientation that mimicked a typical patient-provider interaction when prescribing a medication, including the rationale for effectiveness and the importance of taking the placebo pills as prescribed. The UCO group only received standardized education on fatigue management and energy conservation. Those randomized to OLP were provided with placebo pills and instructed to take two pills, twice a day. OLP adherence was assessed using pill counts. We asked all participants to maintain current treatment regimens over the course of the study. After 21 days, all participants completed final assessments. Participants attended virtual baseline and final assessments at 21 days with two follow-up surveys delivered via email on days 28 and 35. Reminder emails were sent to non-responsive participants for up to day 39 of the study. Participants received $40 in compensation at the end of the study. We will consider the study to be feasible based on set progression criteria of, enrolling the target sample of 48, achieving a retention target of > 80% participants and OLP adherence target of > 90%.

#### Sample size justification

The recommended sample size per arm for pilot feasibility studies is 12. For parallel arm trials, there is a marked gain in precision per increase of 1 in the sample size for small samples but these gains are less pronounced by the point where sample size per arm has reached 12 [24]. Other estimates suggest that an acceptably precise estimate of the standard deviation requires the pilot study itself to be of sufficient size and a sample size of 30 is acceptable size for a pilot study [25]. Further, using 80% upper confidence limit approach, it has been concluded that pilot study sample sizes between 20 and 40 minimizes the overall main study sample size of 80–250 corresponding to effect sizes of 0.4 and 0.7 at 90% power [26]. We therefore extrapolated the recommended per arm sample size of 12 to 16 per arm to account for potential attrition. For 80% powered main trails, per arm pilot trial sample sizes between 10 and 20 will allow for the estimation of small-medium standardized differences [27]. Setting a pilot trial such that it minimizes the sample size for the pilot and main trial is considered the most appropriate method of sample size estimation [21]. Finally for our retention targets using the Wilson score interval, for an 80% retention rate the 95% CI is 67%, 89%.

### Study measures and outcomes

Demographic information collected during the initial screening telephone call included age, race, gender, MS diagnosis date, MS treatments, total number of medications, presence of a diagnosed cognitive or psychiatric disorder, mood disorder, and use of disease modifying therapies.

Primary study measures included feasibility endpoints comprising of recruitment numbers, communication between study coordinators and participants, and other feasibility metrics including median time interval per visit, intervention feasibility, and any adverse events.

Secondary outcome aimed at preliminary assessing effectiveness was fatigue, assessed via the Fatigue Severity Scale (FSS) [28] and the Modified Fatigue Impact Scale (MFIS) [29]. Additional secondary outcomes included the Epworth Sleepiness Scale (ESS) [30], the EuroQOL (quality of life) [31], the Godin Leisure-Time Exercise Questionnaire [32], the Perceived Deficits Questionnaire (PDQ), which assesses cognitive dysfunction [33], the PROMIS Self-Efficacy for Managing the Chronic Disease Scale [34], and the Medical Outcomes Study—Short Form (SF-36) physical and mental component score [35].

### Statistical analysis

Descriptive statistics are provided for feasibility endpoints including recruitment numbers, proportions indicating communication attempts between the coordinators and the participants, median time interval between the survey first started and the end time when the survey was submitted, and reporting of any adverse events. For the secondary analyses, we provide descriptive summary statistics along with estimates from crude linear mixed models (LMM) [36] accounting for participant level heterogeneity, to model the association between the treatment groups and the change in outcome measures at the assessment points versus baseline. We therefore report estimates for mean change at days 21, 28, and 35 with respect to baseline comparing the study groups and provide the 95%, 85%, and 75% confidence intervals, to account for the uncertainty in estimation [21]. Since pilot studies are underpowered for a conventional 5% significance threshold, research recommends that, if the primary purpose of a pilot study is to provide preliminary evidence of the efficacy of an intervention, the significance level can be increased for confidence intervals and for the purpose of interval estimation [21]. Accordingly, for preliminary testing in pilot trials up to a (one sided) *α* = 0.25 has been proposed. Note that, for pilot studies, the impact of a type I error is different. While for a definitive study, type I error would mean that treatments would falsely be concluded as beneficial, for a pilot study, the impact of a type I error is that a definitive study may falsely be undertaken [21].

All analyses were performed using the statistical software SAS 9.4 [37].

## Results

### Feasibility endpoints

#### Recruitment numbers (consort)

**Fig. 1:**
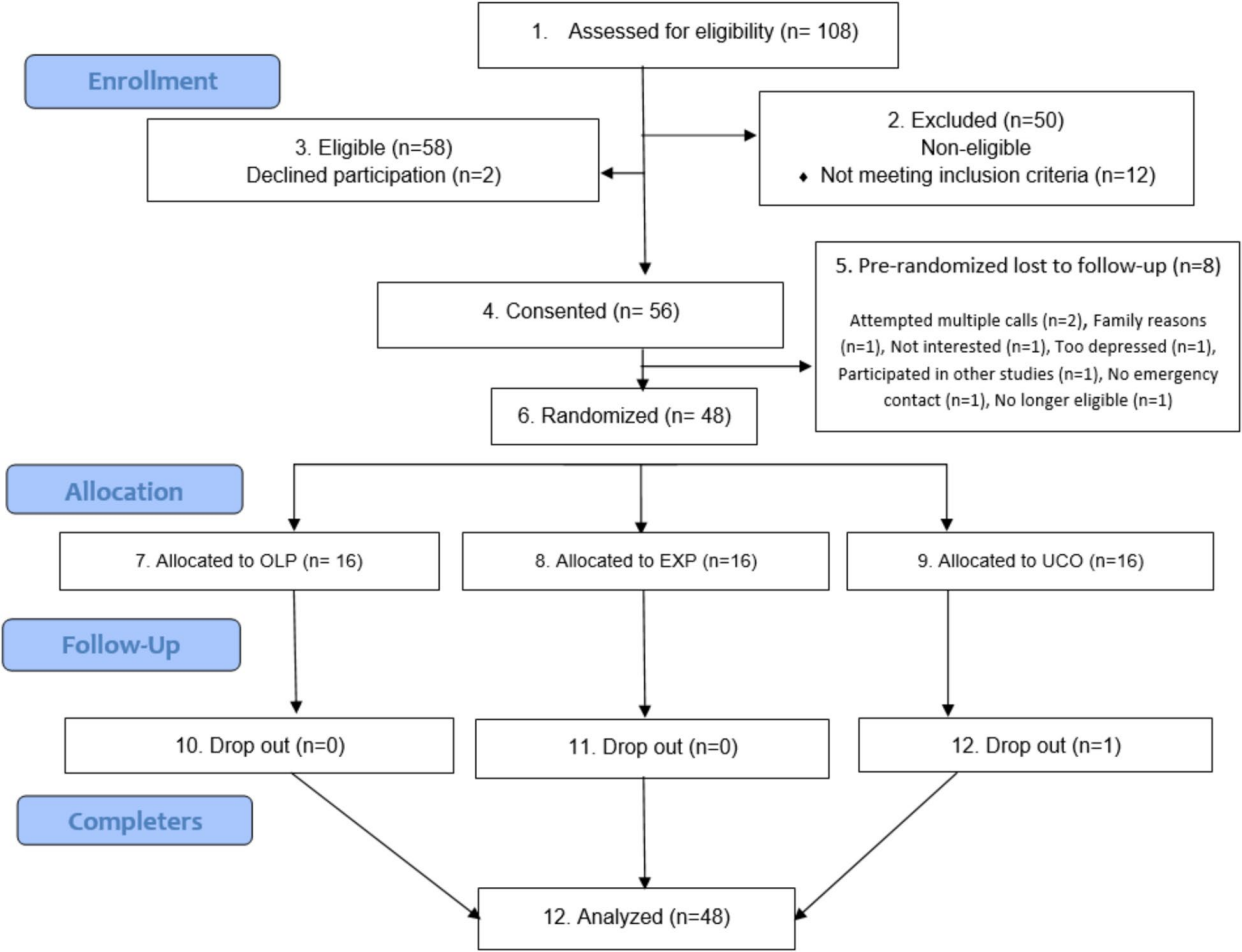
CONSORT flow diagram depicting participant flow

 See Fig. 1 for CONSORT flow diagram. Overall, 108 participants were screened for this study. We excluded 50 participants and 12 did not meet inclusion criteria. Of the 58 eligible, 2 refused to participate. Finally, 56 consented to participate in the study, of which 8 were lost to follow-up pre-randomization. This study consisted of 48 participants who were then randomized to each of the three study groups, with 16 per group.

#### Communication

At the screening visit, our coordinators reached out to 58 participants: 46 (79.31%) of them were contacted once and others were contacted twice to schedule the baseline visit. For the mid-point day 11 follow-up, 29 participants were contacted with the majority of them (93.10%) receiving at least one call. Lastly, to schedule day 21 visit, 32 participants were reached, and 30 (93.75%) of them were able to schedule their next visit in a phone call. Next, 39 participants completed the survey at baseline, 19 (48.72%) of them completed within 2 days after receiving the invite, 15 received one reminder, and one received 6 reminders. For the day 21 survey, 32 participants completed the survey with 19 (59.38%) of them completing it within 2 days. A total of 28 participants completed day 28 survey, and 18 (64%) of those completed without any further reminders. Lastly, for day 35 visit, 29 participants completed the survey; 22 (75.86%) completed without any additional reminder.

#### Feasibility measures

The median time interval for the baseline visit was 35 min, and 21 min for the day 21 survey. Participants spent around 17.5 min for the day 28 and 18.5 min for the day 35 survey. Intervention feasibility—day 11 contact for all three arms and treatment acceptability survey at day 21 (for OLP arm): At the day 11 visit, all three arms of participants were contacted with tailored questions for their specific intervention. Among the OLP arm participants, 13 reported taking the pills for all 11 days, one reported taking it for 9 days, and one participant reported the they “just forgot” to take the pill in the past 11 days. While all OLP participants reported to use the strategies provided in the educational material to reduce fatigue every day, 81.0% of the non-OLP participants reported to use strategies every day, 14.3% used strategies at least once for 5 or more days, and 4.8% used at least once for 2 to 4 days. Further, 77.8% (7 out of 9 in OLP group, assessed at day 21) of the OLP participants considered prescribing placebo as a medication to treat fatigue moderately to completely acceptable, and that it is acceptable to prescribe placebo as a medication if it could enhance the efficacy of the usual fatigue.

#### Adverse event

One mild adverse event was reported and determined as clearly unrelated to the study as the hospitalization happened before the intervention started.

Further, Table 1 provides baseline descriptive statistics for the study participants. Table shows that the mean age for the three study groups was around 50 years and majority (> 80%) were females. While respectively there were about 31% and 25% Blacks in OLP and EXP group, their proportion in UCO group was 50%. Over 85% in each group had a relapsing–remitting type of MS.

**Table 1:** Descriptive statistics for demographic variables by randomization group

Covariates	Mean (SD)/frequencies (%)		
	OLP (*N* = 16)	EXP (*N* = 16)	UCO (*N* = 16)
Age (years)	49.67 (8.25)	53.69 (10.69)	48.53 (11.24)
Sex			
Male	3 (18.75)	2 (12.50)	3 (18.75)
Female	13 (81.25)	14 (87.50)	13 (81.25)
Race			
White	11 (68.75)	11 (68.75)	8 (50.00)
Black/African American	5 (31.25)	4 (25.00)	8 (50.00)
Other	0 (0.00)	1 (6.25)	0 (0.00)
PDDS			
0	2 (11.76)	1 (6.25)	3 (18.75)
1	2 (11.76)	2 (12.50)	4 (25.00)
2	2 (11.76)	4 (25.00)	0 (0.00)
3	4 (23.53)	3 (18.75)	3 (18.75)
4	4 (23.53)	3 (18.75)	1 (6.25)
5	0 (0.00)	0 (0.00)	1 (6.25)
6	2 (11.76)	2 (12.50)	1 (6.25)
7	1 (5.88)	1 (6.25)	2 (12.50)
MS type			
Progressive	0 (0.00)	0 (0.00)	2 (12.50)
Relapsing–remitting	15 (88.24)	14 (87.50)	14 (87.50)
Unknown	2 (11.76)	2 (12.50)	0 (0.00)

*SD*, Standard deviation

In general, FSS in the three groups were over 5 at all assessment days. Descriptively, the MFIS total scores for all the three groups decreased until day 28 post baseline (Table 2). Table S1 provides summary statistics for secondary outcome measures.

**Table 2:** Means and standard deviations for study measures by randomization group and assessment points

Outcomes	Mean (SD)											
	OLP				EXP				UCO			
	Baseline	Day 21	Day 28	Day 35	Baseline	Day 21	Day 28	Day 35	Baseline	Day 21	Day 28	Day 35
FSS mean score	5.74 (0.65)	5.49 (0.75)	5.34 (0.8)	5.44 (0.86)	5.83 (0.91)	5.43 (0.83)	5.52 (0.79)	5.63 (0.95)	5.07 (1.52)	5.48 (1.35)	5.11 (1.4)	5.49 (1.13)
MFIS Physical Subscale	23.65 (7.35)	20.63 (8.33)	19.43 (8.45)	21.31 (6.65)	25.25 (7.59)	21.93 (6.7)	20.15 (6.87)	23 (8.16)	23.67 (6.95)	22.47 (8.4)	21.5 (8.24)	22.53 (7.57)
MFIS Cognitive Subscale	19.35 (9.02)	15.94 (7.51)	16 (7.37)	17.88 (7.7)	23.81 (10.41)	20.07 (9.81)	18.15 (9.16)	21.13 (10.05)	20.93 (8.06)	20.53 (10.3)	19.5 (10.18)	19.67 (9.83)
MFIS Psychosocial Subscale	4.35 (2.15)	4.06 (2.02)	4 (2.29)	4 (1.9)	5.31 (1.99)	4.57 (2.03)	3.54 (1.51)	4.38 (2.03)	5.07 (2.28)	4.4 (2.16)	3.75 (1.96)	4.53 (2.07)
MFIS total score	47.35 (16.15)	40.63 (15.68)	39.43 (14.67)	43.19 (13.9)	54.38 (17.17)	46.57 (17.63)	41.85 (16.17)	48.5 (19.66)	49.67 (13.53)	47.4 (17.37)	44.75 (17.85)	46.73 (17.31)

*OLP* Open label placebo (i.e., prescription to take 2 placebo pills, twice a day) along with conveying a positive expectancy for placebo effects, *EXP* Positive expectancy for placebo effects group that kept a breathing log and were asked to breathe for 1 min in the morning and evening, *UCO* Usual care only (i.e., standard care and educational materials), *SD* Standard deviation

Table 3 provides secondary analyses based estimates from the crude LMM analyzing the change in FSS mean scores. At day 21 with respect to baseline, the change in fatigue in OLP and EXP group versus UCO group was about 0.6 units lower (95% CI: − 1.206, − 0.003; − 1.301, − 0.065, respectively). For the same, the change at 28 and 35 days was respectively 0.5 and 0.4 (− 1.145, 0.138; − 1.057, 0.245) and 0.7 and 0.6 units lower (− 1.308, − 0.114; − 1.224, − 0.023). Estimates and CIs for additional secondary outcomes are presented in Table S2.

**Table 3:** Crude LMM comparing change in fatigue scores from baseline among the randomization groups

Outcome	Comparison group	Assessment day versus baseline (change in score)	Estimate	95% CI	85% CI	75% CI
FSS mean score	OLP vs. EXP	21	0.078	(− 0.523, 0.679)	(− 0.362, 0.518)	(− 0.273, 0.429)
		28	− 0.097	(− 0.721, 0.527)	(− 0.554, 0.359)	(− 0.461, 0.267)
		35	− 0.087	(− 0.675, 0.500)	(− 0.517, 0.343)	(− 0.430, 0.256)
	OLP vs. UCO	21	− 0.605	(− 1.206, − 0.003)	(− 1.045, − 0.164)	(− 0.956, − 0.253)
		28	− 0.503	(− 1.145, 0.138)	(− 0.973, − 0.034)	(− 0.878, − 0.129)
		35	− 0.711	(− 1.308, − 0.114)	(− 1.148, − 0.274)	(− 1.060, − 0.362)
	EXP vs. UCO	21	− 0.683	(− 1.301, − 0.065)	(− 1.135, − 0.230)	(− 1.044, − 0.322)
		28	− 0.406	(− 1.057, 0.245)	(− 0.882, 0.070)	(− 0.786, − 0.026)
		35	− 0.624	(− 1.224, − 0.023)	(− 1.063, − 0.184)	(− 0.974, − 0.273)
MFIS Physical Subscale	OLP vs. EXP	21	0.383	(− 2.752, 3.519)	(− 1.911, 2.678)	(− 1.447, 2.214)
		28	0.61	(− 2.647, 3.866)	(− 1.774, 2.993)	(− 1.292, 2.511)
		35	0.056	(− 3.007, 3.119)	(− 2.186, 2.298)	(− 1.733, 1.845)
	OLP vs. UCO	21	− 1.366	(− 4.507, 1.774)	(− 3.665, 0.932)	(− 3.200, 0.467)
		28	− 1.122	(− 4.477, 2.233)	(− 3.577, 1.333)	(− 3.081, 0.837)
		35	− 1.061	(− 4.175, 2.054)	(− 3.340, 1.218)	(− 2.879, 0.758)
	EXP vs. UCO	21	− 1.750	(− 4.978, 1.478)	(− 4.112, 0.612)	(− 3.635, 0.135)
		28	− 1.732	(− 5.135, 1.671)	(− 4.222, 0.759)	(− 3.719, 0.255)
		35	− 1.117	(− 4.249, 2.015)	(− 3.409, 1.175)	(− 2.945, 0.712)
MFIS Cognitive Subscale	OLP vs. EXP	21	0.331	(− 3.284, 3.946)	(− 2.314, 2.977)	(− 1.780, 2.442)
		28	1.477	(− 2.279, 5.232)	(− 1.272, 4.225)	(− 0.716, 3.669)
		35	1.52	(− 2.011, 5.052)	(− 1.064, 4.105)	(− 0.542, 3.583)
	OLP vs. UCO	21	− 2.582	(− 6.203, 1.040)	(− 5.232, 0.068)	(− 4.696, − 0.467)
		28	− 0.315	(− 4.184, 3.554)	(− 3.146, 2.516)	(− 2.574, 1.944)
		35	0.1	(− 3.491, 3.691)	(− 2.528, 2.727)	(− 1.997, 2.196)
	EXP vs. UCO	21	− 2.913	(− 6.635, 0.809)	(− 5.637, − 0.189)	(− 5.086, − 0.740)
		28	− 1.791	(− 5.716, 2.133)	(− 4.664, 1.081)	(− 4.083, 0.500)
		35	− 1.421	(− 5.032, 2.190)	(− 4.063, 1.222)	(− 3.529, 0.688)
MFIS Psychosocial Subscale	OLP vs. EXP	21	0.628	(− 0.476, 1.731)	(− 0.180, 1.435)	(− 0.017, 1.272)
		28	1.194	(0.049, 2.340)	(0.356, 2.033)	(0.525, 1.863)
		35	0.629	(− 0.450, 1.707)	(− 0.160, 1.418)	(− 0.001, 1.259)
	OLP vs. UCO	21	0.399	(− 0.706, 1.503)	(− 0.410, 1.207)	(− 0.247, 1.044)
		28	0.957	(− 0.222, 2.136)	(0.094, 1.820)	(0.269, 1.645)
		35	0.225	(− 0.872, 1.321)	(− 0.578, 1.027)	(− 0.416, 0.865)
	EXP vs. UCO	21	− 0.229	(− 1.365, 0.906)	(− 1.060, 0.602)	(− 0.892, 0.434)
		28	− 0.237	(− 1.433, 0.959)	(− 1.113, 0.638)	(− 0.936, 0.461)
		35	− 0.404	(− 1.507, 0.699)	(− 1.211, 0.403)	(− 1.048, 0.240)
MFIS total score	OLP vs. EXP	21	1.355	(− 5.348, 8.058)	(− 3.550, 6.260)	(− 2.559, 5.268)
		28	3.267	(− 3.696, 10.230)	(− 1.829, 8.362)	(− 0.799, 7.332)
		35	2.207	(− 4.342, 8.756)	(− 2.585, 6.999)	(− 1.617, 6.031)
	OLP vs. UCO	21	− 3.55	(− 10.265, 3.164)	(− 8.464, 1.363)	(− 7.471, 0.370)
		28	− 0.487	(− 7.659, 6.686)	(− 5.735, 4.762)	(− 4.675, 3.701)
		35	− 0.735	(− 7.393, 5.923)	(− 5.607, 4.138)	(− 4.622, 3.153)
	EXP vs. UCO	21	− 4.905	(− 11.806, 1.996)	(− 9.955, 0.145)	(− 8.935, − 0.876)
		28	− 3.753	(− 11.030, 3.523)	(− 9.078, 1.571)	(− 8.002, 0.495)
		35	− 2.942	(− 9.637, 3.754)	(− 7.841, 1.958)	(− 6.851, 0.968)

*OLP* Open label placebo (i.e., prescription to take 2 placebo pills, twice a day) along with conveying a positive expectancy for placebo effects, *EXP* Positive expectancy for placebo effects group that kept a breathing log and were asked to breathe for 1 min in the morning and evening, *UCO* Usual care only (i.e., standard care and educational materials), *CI* Confidence interval

## Discussion

This pilot study is the first effort to assess the feasibility of using an OLP to address MS fatigue. We provided preliminary effect estimates comparing the OLP group with the EXP and UCO as well as the EXP group with the UCO. We also aimed to determine whether the act of taking medication, as opposed to ritual of performing breathing exercises, changes the outcomes of interests.

Of the 108 participants screened for this study, 48 were randomized to the three study groups equally, meeting our eligibility criteria. At day 21, about 7/9 available of the OLP participants considered prescribing placebo as a medication to treat fatigue moderately to completely acceptable, and that it is acceptable to prescribe placebo as a medication if it could enhance the efficacy of the usual fatigue. This is in line with existing qualitative studies [38, 39] reporting that a majority of the patients are willing to use OLPs if prescribed by a doctor. Patients have overall indicated that the relationship with their provider would not be impacted by being prescribed placebos while also expressing skepticism about the effectiveness of OLPs initially. It has been concluded that while patients may experience some reluctance to taking OLPs, they may not be resistant to them being a treatment option offered in a clinical setting if prescribed by a medical provider [38].

Next, LMM based estimates showed that the OLP group though had marginally (0.08–0.09) lower FSS as compared to EXP group at days 28 and 35 (95% CI: − 0.721, 0.527; − 0.675, 0.500, respectively). As compared to the UCO also, the OLP group had 0.5–0.7 units lower FSS at all the assessment days (day 21: − 1.206, − 0.003; day 28: − 1.145, 0.138; day 35: − 1.308, − 0.114). Previous research [11] efforts have concluded that there may be potential therapeutic benefits of OLP and that placebo and medication effects can be modulated by expectancies [11]. Another RCT [18] that investigated the effect of OLP on cancer-related fatigue in survivors and assessed the biologic and psychological correlates of placebo efficacy demonstrated that at days 8 and 22 post baseline, OLP group was found to have a significant improvement in fatigue, while the control group did not. Another study [13] that assessed if for patients experiencing chronic low back pain, placebo effects could be harnessed ethically by adding OLP treatment to treatment as usual (TAU) for 3 weeks reported that in comparison to TAU, OLP group had greater pain reduction (1.5, 0.8–2.3) and reduced disability (3.4, 2.2–4.5).

### Limitations

The estimates provided in this study should be interpreted with caution as these are based on the sample size of this pilot study. While participants could not be blinded, the statistician could have been blinded.

## Conclusions

Our study protocol piloting OLP to treat MS-related fatigue met the feasibility criteria of recruitment and retention. Participants in this study generally reported OLP to be acceptable and that it may be prescribed as a “medication” if it could benefit their MS fatigue severity. We assessed preliminary evidence on the potential effectiveness of treatment group assignment on MS-related fatigue and other outcomes and future confirmatory study will be needed to test the efficacy of OLP in treating MS-related fatigue, especially larger trials of OLP which manipulate expectancies (e.g., neutral vs. positive) and practitioner-patient relationship factors (e.g., empathy, active listening) to determine whether OLP might be a viable treatment for MS fatigue.

## Supplementary Information

Additional file 1: Supplementary tables.

## Data Availability

The datasets used and/or analyzed during the current study are available from the corresponding author on reasonable request.

## Abbreviations

MS: Multiple sclerosis
OLP: Open-label placebo
OLP: Open label placebo (i.e., prescription to take 2 placebo pills, twice a day) along with conveying a positive expectancy for placebo effects
EXP: Positive expectancy for placebo effects group that kept a breathing log and were asked to breathe for 1 min in the morning and evening
UCO: Usual care only
FSS: Fatigue Severity Scale
MFIS: Modified Fatigue Impact Scale
LMM: Linear mixed models
CRF: Cancer-related fatigue
SD: Standard deviation
CI: Confidence interval
TAU: Treatment as usual
