# Supplementary material for: Open label placebo to treat fatigue in people with multiple sclerosis: feasibility and preliminary effects
Source: Pilot Feasibility Stud. 2025 Jul 3;11:93. doi: 10.1186/s40814-025-01674-w (PMC12226847; doi:10.1186/s40814-025-01674-w)
Supplement: Supplementary file 1 — Additional file 1: Supplementary tables. [file 40814_2025_1674_MOESM1_ESM.docx]

**Supplementary Tables**

**Table S.1: Means and Standard Deviations for study measures by randomization group and assessment day**

| **Outcomes** | **Means (SD)** | | | | | | | | | | | | |
| --- | --- | --- | --- | --- | --- | --- | --- | --- | --- | --- | --- | --- | --- |
|  | **OLP** | | | | **EXP** | | | | **UCO** | | | | |
|  | **Baseline** | **Day** **21** | **Day** **28** | **Day** **35** | **Baseline** | **Day** **21** | **Day** **28** | **Day** **35** | **Baseline** | **Day** **21** | **Day** **28** | **Day** **35** |  |
| ESS sum score | 8.06 (4.6) | 7.31 (3.86) | 6.07 (3.63) | 7.13 (3.46) | 10.06 (5.42) | 9.86 (5.76) | 8.38 (5.44) | 9 (4.65) | 8.93 (4.32) | 8.4 (5.63) | 8.25 (5.51) | 8.67 (4.69) |  |
| EuroQoL | 0.61 (0.09) | 0.61 (0.09) | 0.63 (0.1) | 0.61 (0.11) | 0.62 (0.08) | 0.62 (0.11) | 0.65 (0.08) | 0.63 (0.1) | 0.61 (0.09) | 0.61 (0.11) | 0.63 (0.11) | 0.62 (0.1) |  |
| Godin total score | 10.59 (9.72) | 14.81 (20.25) | 11.64 (10.04) | 11.06 (10.11) | 10.88 (21.32) | 19.79 (36.3) | 14.85 (22.56) | 21.31 (33.41) | 20.73 (20.59) | 23.33 (19.21) | 32.33 (34.01) | 20.87 (17.94) |  |
| PDQ sum score | 8.88 (3.44) | 8.44 (3.52) | 7.86 (3.13) | 7.75 (2.96) | 12.5 (4.1) | 11.14 (4.17) | 9.77 (3.37) | 10.56 (3.5) | 10.67 (4.67) | 10.8 (4.81) | 9.17 (5.08) | 9.13 (4.84) |  |
| PROMIS Physical Health | 38.36 (6.82) | 38.7 (7.72) | 40.56 (7.72) | 38.94 (8.94) | 39.66 (7.41) | 40.42 (8.04) | 42.41 (6.23) | 39.63 (7.6) | 39.32 (6.74) | 38.51 (8.82) | 40.23 (8.39) | 39.77 (7.83) |  |
| PROMIS Mental Health | 42.66 (5.59) | 42.34 (7.77) | 43.46 (6.02) | 43.5 (5.38) | 43.33 (7.11) | 42.18 (8.97) | 47.12 (6.75) | 44.94 (8.62) | 42.51 (6.8) | 45.1 (7.1) | 44.93 (8.88) | 45 (6.6) |  |
| SF-36 PCS | 34.21 (11.23) | 35.77 (10.28) | 37.27 (9.88) | 36.03 (11.06) | 33.6 (13.12) | 40.21 (10.59) | 37.39 (14.37) | 37.55 (11.1) | 36.12 (12.82) | 34.27 (11.02) | 35.59 (12.3) | 35.09 (11.56) |  |
| SF-36 MCS | 38.47 (14.03) | 40.88 (13.87) | 40.98 (13.99) | 40.24 (12.26) | 43.9 (13.36) | 38.26 (16.22) | 46.16 (14.66) | 39.91 (14.4) | 34.99 (8.44) | 40.91 (14.28) | 41.7 (13.46) | 39.78 (15.37) |  |

OLP: Open Label Placebo (i.e., prescription to take 2 placebo pills, twice a day) along with conveying a positive expectancy for placebo effects; EXP: positive expectancy for placebo effects group that kept a breathing log and were asked to breathe for one minute in the morning and evening; UCO: Usual Care Only (i.e., standard care and educational materials)

SD: Standard Deviation

**Table S.2: Crude LMM comparing change in fatigue scores from baseline among the randomization groups**

| **Outcome** | **Comparison Group** | **Assessment day versus baseline (Change in score)** | **Estimate** | **95% CI** | **85% CI** | **75% CI** |
| --- | --- | --- | --- | --- | --- | --- |
| ESS sum score | OLP vs EXP | 21 | -0.262 | (-2.154, 1.629) | (-1.646, 1.122) | (-1.367, 0.842) |
|  |  | 28 | 0.242 | (-1.723, 2.207) | (-1.196, 1.680) | (-0.905, 1.389) |
|  |  | 35 | 0.208 | (-1.640, 2.055) | (-1.145, 1.560) | (-0.871, 1.286) |
|  | OLP vs UCO | 21 | 0.667 | (-1.228, 2.561) | (-0.720, 2.053) | (-0.440, 1.773) |
|  |  | 28 | -0.353 | (-2.377, 1.671) | (-1.834, 1.128) | (-1.535, 0.829) |
|  |  | 35 | -0.588 | (-2.467, 1.290) | (-1.963, 0.787) | (-1.685, 0.509) |
|  | EXP vs UCO | 21 | 0.929 | (-1.018, 2.876) | (-0.496, 2.354) | (-0.208, 2.066) |
|  |  | 28 | -0.595 | (-2.648, 1.459) | (-2.097, 0.908) | (-1.793, 0.604) |
|  |  | 35 | -0.796 | (-2.685, 1.093) | (-2.178, 0.587) | (-1.899, 0.307) |
| EuroQoL | OLP vs EXP | 21 | -0.002 | (-0.039, 0.035) | (-0.029, 0.025) | (-0.024, 0.019) |
|  |  | 28 | -0.003 | (-0.041, 0.036) | (-0.030, 0.025) | (-0.025, 0.020) |
|  |  | 35 | -0.003 | (-0.039, 0.033) | (-0.029, 0.023) | (-0.024, 0.018) |
|  | OLP vs UCO | 21 | -0.009 | (-0.046, 0.028) | (-0.036, 0.018) | (-0.031, 0.012) |
|  |  | 28 | -0.01 | (-0.049, 0.029) | (-0.039, 0.019) | (-0.033, 0.013) |
|  |  | 35 | -0.014 | (-0.051, 0.022) | (-0.041, 0.012) | (-0.036, 0.007) |
|  | EXP vs UCO | 21 | -0.007 | (-0.045, 0.031) | (-0.035, 0.021) | (-0.029, 0.015) |
|  |  | 28 | -0.008 | (-0.047, 0.032) | (-0.037, 0.022) | (-0.031, 0.016) |
|  |  | 35 | -0.012 | (-0.048, 0.025) | (-0.038, 0.015) | (-0.033, 0.010) |
| Godin total score | OLP vs EXP | 21 | -4.096 | (-16.489, 8.297) | (-13.165, 4.973) | (-11.332, 3.140) |
|  |  | 28 | -5.963 | (-18.830, 6.904) | (-15.379, 3.453) | (-13.476, 1.550) |
|  |  | 35 | -9.84 | (-21.952, 2.272) | (-18.704, -0.976) | (-16.912, -2.768) |
|  | OLP vs UCO | 21 | 5.518 | (-6.892, 17.928) | (-3.563, 14.600) | (-1.728, 12.764) |
|  |  | 28 | -5.641 | (-18.883, 7.602) | (-15.332, 4.051) | (-13.373, 2.092) |
|  |  | 35 | 0.464 | (-11.851, 12.779) | (-8.548, 9.476) | (-6.726, 7.655) |
|  | EXP vs UCO | 21 | 9.614 | (-3.138, 22.366) | (0.282, 18.946) | (2.168, 17.060) |
|  |  | 28 | 0.323 | (-13.112, 13.757) | (-9.509, 10.154) | (-7.522, 8.167) |
|  |  | 35 | 10.304 | (-2.081, 22.689) | (1.241, 19.367) | (3.073, 17.535) |
| PDQ sum score | OLP vs EXP | 21 | 0.646 | (-1.051, 2.343) | (-0.596, 1.888) | (-0.345, 1.637) |
|  |  | 28 | 1.277 | (-0.486, 3.040) | (-0.013, 2.567) | (0.248, 2.306) |
|  |  | 35 | 0.849 | (-0.809, 2.507) | (-0.365, 2.062) | (-0.119, 1.817) |
|  | OLP vs UCO | 21 | -0.37 | (-2.070, 1.330) | (-1.614, 0.874) | (-1.363, 0.622) |
|  |  | 28 | 1.194 | (-0.622, 3.009) | (-0.135, 2.522) | (0.133, 2.254) |
|  |  | 35 | 0.445 | (-1.241, 2.131) | (-0.789, 1.679) | (-0.540, 1.429) |
|  | EXP vs UCO | 21 | -1.016 | (-2.763, 0.731) | (-2.295, 0.263) | (-2.036, 0.004) |
|  |  | 28 | -0.083 | (-1.926, 1.759) | (-1.432, 1.265) | (-1.159, 0.992) |
|  |  | 35 | -0.404 | (-2.100, 1.291) | (-1.645, 0.837) | (-1.394, 0.586) |
| PROMIS Physical Health | OLP vs EXP | 21 | -0.576 | (-3.239, 2.088) | (-2.525, 1.374) | (-2.131, 0.980) |
|  |  | 28 | -0.671 | (-3.438, 2.097) | (-2.696, 1.355) | (-2.287, 0.945) |
|  |  | 35 | 0.202 | (-2.400, 2.804) | (-1.702, 2.106) | (-1.317, 1.721) |
|  | OLP vs UCO | 21 | -0.006 | (-2.675, 2.662) | (-1.959, 1.947) | (-1.565, 1.552) |
|  |  | 28 | -0.145 | (-2.997, 2.706) | (-2.232, 1.942) | (-1.810, 1.520) |
|  |  | 35 | -0.27 | (-2.915, 2.376) | (-2.206, 1.666) | (-1.815, 1.275) |
|  | EXP vs UCO | 21 | 0.569 | (-2.174, 3.312) | (-1.438, 2.577) | (-1.032, 2.171) |
|  |  | 28 | 0.525 | (-2.368, 3.418) | (-1.592, 2.642) | (-1.164, 2.214) |
|  |  | 35 | -0.472 | (-3.132, 2.189) | (-2.419, 1.475) | (-2.025, 1.082) |
| PROMIS Mental Health | OLP vs EXP | 21 | -1.002 | (-4.019, 2.015) | (-3.210, 1.206) | (-2.764, 0.760) |
|  |  | 28 | -1.557 | (-4.691, 1.577) | (-3.851, 0.737) | (-3.387, 0.273) |
|  |  | 35 | -1.253 | (-4.201, 1.695) | (-3.410, 0.905) | (-2.974, 0.469) |
|  | OLP vs UCO | 21 | -3.467 | (-6.489, -0.444) | (-5.679, -1.255) | (-5.232, -1.702) |
|  |  | 28 | -2.62 | (-5.848, 0.609) | (-4.982, -0.257) | (-4.505, -0.735) |
|  |  | 35 | -2.133 | (-5.130, 0.864) | (-4.327, 0.060) | (-3.883, -0.383) |
|  | EXP vs UCO | 21 | -2.465 | (-5.571, 0.642) | (-4.738, -0.191) | (-4.278, -0.651) |
|  |  | 28 | -1.063 | (-4.338, 2.213) | (-3.459, 1.334) | (-2.975, 0.850) |
|  |  | 35 | -0.880 | (-3.895, 2.134) | (-3.086, 1.325) | (-2.640, 0.879) |
| SF 36 PCS | OLP vs EXP | 21 | -3.029 | (-7.390, 1.333) | (-6.220, 0.163) | (-5.575, -0.482) |
|  |  | 28 | -0.464 | (-4.995, 4.067) | (-3.780, 2.852) | (-3.110, 2.182) |
|  |  | 35 | -2.236 | (-6.497, 2.025) | (-5.354, 0.882) | (-4.724, 0.252) |
|  | OLP vs UCO | 21 | 2.713 | (-1.657, 7.082) | (-0.485, 5.910) | (0.161, 5.264) |
|  |  | 28 | 3.437 | (-1.232, 8.105) | (0.020, 6.853) | (0.711, 6.163) |
|  |  | 35 | 2.752 | (-1.580, 7.085) | (-0.418, 5.923) | (0.223, 5.282) |
|  | EXP vs UCO | 21 | 5.741 | (1.250, 10.232) | (2.455, 9.028) | (3.119, 8.364) |
|  |  | 28 | 3.900 | (-0.836, 8.637) | (0.435, 7.366) | (1.135, 6.666) |
|  |  | 35 | 4.988 | (0.632, 9.345) | (1.800, 8.177) | (2.445, 7.532) |
| SF 36 MCS | OLP vs EXP | 21 | 5.501 | (-2.540, 13.542) | (-0.383, 11.386) | (0.806, 10.196) |
|  |  | 28 | 0.716 | (-7.630, 9.063) | (-5.391, 6.824) | (-4.157, 5.590) |
|  |  | 35 | 5.077 | (-2.783, 12.937) | (-0.675, 10.829) | (0.488, 9.666) |
|  | OLP vs UCO | 21 | -4.583 | (-12.634, 3.467) | (-10.474, 1.308) | (-9.284, 0.117) |
|  |  | 28 | -6.708 | (-15.294, 1.878) | (-12.992, -0.425) | (-11.722, -1.695) |
|  |  | 35 | -3.703 | (-11.695, 4.289) | (-9.551, 2.145) | (-8.369, 0.963) |
|  | EXP vs UCO | 21 | -10.084 | (-18.356, -1.812) | (-16.137, -4.031) | (-14.914, -5.254) |
|  |  | 28 | -7.425 | (-16.135, 1.286) | (-13.799, -1.050) | (-12.511, -2.339) |
|  |  | 35 | -8.780 | (-16.817, -0.742) | (-14.662, -2.898) | (-13.473, -4.087) |

OLP: Open Label Placebo (i.e., prescription to take 2 placebo pills, twice a day) along with conveying a positive expectancy for placebo effects; EXP: positive expectancy for placebo effects group that kept a breathing log and were asked to breathe for one minute in the morning and evening; UCO: Usual Care Only (i.e., standard care and educational materials)

CI: Confidence Interval
